# Cardiovascular disease in older people with serious mental illness: Current challenges and future directions

**DOI:** 10.3389/fpsyt.2023.1110361

**Published:** 2023-02-28

**Authors:** Katherine Chin, Sudip Ghosh, Hari Subramaniam, Lucy Beishon

**Affiliations:** ^1^Department of Ageing and Health, Guy’s and St Thomas’ Hospital, London, United Kingdom; ^2^Leicester School of Allied Health Sciences, De Montfort University, Leicester, United Kingdom; ^3^The Evington Centre, Leicestershire Partnership National Health Service (NHS) Trust, Leicester, United Kingdom; ^4^Department of Cardiovascular Sciences, University of Leicester, Leicester, United Kingdom; ^5^National Institute for Health Research Leicester Biomedical Research Centre, British Heart Foundation Cardiovascular Research Centre, Glenfield Hospital, Leicester, United Kingdom

**Keywords:** ischaemic heart disease, mental health, acute coronary syndrome, chronic heart disease, aged, cardiovascular

## Introduction

By 2050, it is projected that the population of over 60 years old will reach 2.1 billion, from 900 million in 2015 (1). A total of 20% of this cohort have a neurological or mental health disorder, which is expected to rise in line with these changing population demographics (2). Anxiety, substance abuse disorders, schizophrenia and bipolar disorder are also seen commonly in older people. Serious mental illness (SMI) is a term used to group several common psychiatric disorders (schizophrenia, bipolar affective disorder and major depressive disorder) which significantly affect functional abilities (3). While the mortality gap remains significant between people living with SMI and the general population, older people with SMI are routinely cared for by old age psychiatrists and have distinct challenges from the younger SMI population (4). These challenges include greater frailty, high levels of physical health morbidity, polypharmacy, and greater levels of cognitive and functional impairments (1). A recent study found that 17.5% of 65–84 year olds have both a physical and mental health condition, rising to ∼30% of over 85 year olds (5). There is a substantial interplay between physical and mental health and people with SMI have a 10–20 year reduction in life expectancy compared to those without (6, 7).

## Cardiovascular disease and SMI

Physical ill health is the most significant factor in the widening mortality gap between SMI and the general population (1). Cardiovascular disease (CVD) is the leading cause of mortality worldwide (8), and the major cause of death in SMI (9), and CVD increases with age (10). The relationship is bidirectional, and those with CVD are also at higher risk of adverse mental health (9). Much of this risk is modifiable through better provision and access to physical healthcare (11). A large meta-analysis of observational studies found higher rates of all CVD sub-types amongst those with SMI, but contemporary data in the UK is lacking (12), the last studies being conducted over a decade ago (13, 14). The average age of participants with SMI was ∼50 years in these studies (12–14), and few studies specifically investigate older populations. One of the major challenges to understanding this relationship is the fragmented nature of physical and mental health services, and the lack of integrated data systems through which to achieve this. Furthermore, interventions need to be tailored for older people given the unique challenges associated with managing CVD in SMI with concomitant frailty, cognitive and physical impairments (15). In the UK, CVD was highlighted as a key priority in the National Health Service (NHS) long term plan (16). Inequality of care for patients with SMI was highlighted, and a toolkit has been developed to improve rates of detection, prevention and treatment in SMI (16).

In a large meta-analysis, patients with SMI had a 53% higher risk of CVD and 85% higher CVD-related mortality compared to the general population (12). However, the majority of these studies focused on younger populations. In a large study of over 600,000 of community dwelling over 65-year-olds with SMI or substance use disorders, there was an increased risk of hypertension, ischaemic heart disease, congestive heart failure, and atrial fibrillation compared to community dwelling older people and those in long-term care (17).

Depression is associated with a greater risk of cardiovascular mortality in older adults (18, 19). There is also an increased risk of coronary heart disease in depressive disorders (20–22). Even mild depression has a higher risk of CVD, which is greater amongst those with chronic or recurrent depression (20). Furthermore, there is an increased risk of stroke and cardiovascular related mortality in older people with depression and comorbid hypertension (23, 24).

## Risk factors for CVD in SMI

There are several reasons that CVD risk is elevated amongst people with SMI relative to the general population (3), and given that age is an independent risk factor for CVD, these risks are only likely to increase further over time. Firstly, there is an increased rate of risk taking behaviors (e.g., smoking, poor diet, sedentary lifestyle) amongst people with SMI (25). A large study of ∼600,000 patients found higher rates of smoking, diabetes and elevated BMI amongst people diagnosed with SMI, although ten year risk in patients aged 40–75 years was similar between those with and without SMI (25). However, those with established CVD were excluded so it is unclear if CVD rates in older people with and without SMI were comparable. Secondly, antipsychotics, mood stabilizers, and some antidepressants have significant adverse metabolic side effects, and poorer CVD outcomes (26, 27). Antipsychotic medications are associated with dyslipidemia, increased insulin resistance, and weight gain (28, 29). In older people, there are specific concerns that both first and second generation antipsychotics may increase the risk of sudden cardiac death in patients with and without dementia (30). Furthermore, antipsychotic use in dementia has been associated with increased risk of both stroke and myocardial infarction (31, 32). One study found comparable CVD risk between first and second generation antipsychotics in older people, although haloperidol and levomepromazine had greater CVD-risk compared to risperidone (30). Coupled with increases in CVD associated with aging, this could significantly worsen CVD prevalence and mortality, although more studies in older people are needed to confirm this. Older people with SMI also experience inequalities in access to physical healthcare (11, 33), and are more likely to be institutionalized earlier (34). Older people experience greater barriers to accessing health services due to financial constraints, physical and mobility impairments, cognitive impairment, and increased dependency (35).

**FIGURE 1:**
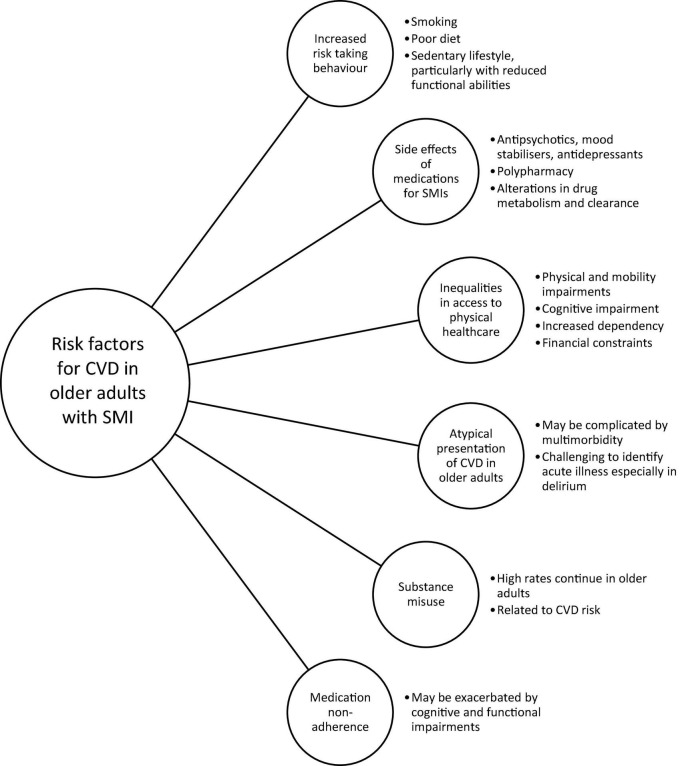
Summary of risk factors for cardiovascular disease (CVD) in older people living with serious mental illness (SMI).

People with SMI also experience significant stigma, are vulnerable, and may have atypical presentations of CVD that are under-recognized by healthcare professionals (36). In older people, presentations can be complicated by the presence of delirium, and recognizing periods of acute illness can be more challenging (37). As a consequence, older people are less likely to be adequately assessed and treated for CVD, which may be compounded by concomitant SMI (36). These factors may also lead to a reduction in access to or engagement with primary and secondary prevention of CVD, such as smoking cessation services, and physical activity programs (3). People with SMI are more likely to have lower socio-economic status (38), with reduced ability to access healthy diets and physical activity services which require subscriptions. Financial barriers are common in older people owing to reduced income, reliance on pensions, and socioeconomic factors (35, 39). Substance misuse is commonly associated with SMI, remains high in older adults (40), and is associated with CVD (17). Rates of medication non-adherence are higher amongst SMI, which may reduce compliance with preventative therapies (e.g., statins, antihypertensives) (41). In older people with cognitive impairment, non-adherence rates may be further exacerbated, and their ability to engage with physical activity programs are more likely to be impaired (4). Figure 1 summarizes the key risk factors for CVD in older people with SMI.

## Current management of CVD in SMI

Interventions to address SMI in CVD can be considered under two main categories. Firstly, primary prevention strategies targeting risk factors and comorbidities, and secondly, secondary prevention strategies targeting individuals who have already experienced CVD (3).

A significant proportion of patients with SMI are managed by primary, as well as secondary care, with around one third being managed solely by primary care (42). However, continuity between primary and secondary care services remains inadequate (42), with significant care inequalities (43). Rates of physical comorbidity for people living with SMI in primary care are high, and increasing over time (44). Therefore, primary care is a key area for the implementation of CVD risk assessment and stratification for older people living with SMI. In England, the quality and outcomes framework (QOF), incentivizes practices to undertake CVD risk assessment (45), and the use of tailored risk assessment calculators for SMI will be particularly important in this setting. However, in older people the balance of risk and care priorities is likely to shift with increasing frailty, and a greater focus on quality of life, requiring a more tailored approach to risk management. Furthermore, primary prevention strategies may be less effective later in life (owing to the long duration required for risk reduction), and these calculators may be less applicable to older populations.

Pharmacological management of SMI in older people is challenging. Older people experience significant polypharmacy (more than or equal to five medications), and can affect up to 96.5% of older people (46). Antipsychotic polypharmacy refers to the use of two or more antipsychotics combined with mood stabilizers, antidepressants, anxiolytics or hypnotics (47). Whilst antipsychotic polypharmacy can benefit some patients (47), older people are more vulnerable to adverse effects owing to alterations in drug metabolism and clearance (46). Furthermore, benzodiazepines, antidepressants, and polypharmacy have been linked to increased risk of falls and hip fracture, but the relationship with antipsychotics is less clear (48). However, approaches which simplify treatment regimens, reduce polypharmacy, and minimize side effects are likely to be beneficial.

In a review by Bartels et al. few interventions have been designed specifically for older people with SMI, despite key differences to younger populations (33). However, integrated care models targeting both physical and mental health using psychosocial skills training, illness self-management, collaborative care, and behavioral health homes have shown benefit (33). An emerging area for future research is the use of digital technology, particularly for older people in long-term care and rural communities. Although research has been limited in this area, telehealth has demonstrated feasibility for treatment monitoring in adults with SMI, which could be used in long-term care facilities (49).

## Discussion

In summary, people living with SMI are at greater risk for CVD than the general population, but how age modulates this risk is less clear. Despite this, rates of assessment and treatment remain low. Older people face a number of distinct challenges compared to younger people living with SMI, which may benefit from a more comprehensive and holistic approach to care. In particular, principles drawn from comprehensive geriatric assessment may be beneficial, even in younger patients who display early signs of frailty and multi-morbidity. Importantly, approach to risk and balance of priorities are likely to differ considerably between younger and older patients with SMI and a personalized approach to risk management, including principles of advanced care planning, will be particularly important for older people living with SMI and frailty. This could be achieved through more integrated care and liaison between physical and mental health practitioners, and primary and secondary care services. Strategies to address both primary and secondary prevention of CVD have shown mixed results, but the emergence of risk calculators, multi-modal programmes, and measures to improve adherence are promising. Much of these interventions may need to be modified to consider challenges associated with aging, particularly in terms of cognitive and functional impairments. More work is needed to elucidate effective ways to manage CVD risk and encourage adherence in older people living with SMI but also to manage long term complications, and promote and enhance recovery through rehabilitation programmes.

## Author contributions

KC, SG, HS, and LB drafted the manuscript. All authors contributed to the article and approved the submitted version.
